# Mutational binding assessment for TCR engineering on experimental and AlphaFold 3 scaffolds

**DOI:** 10.3389/fbinf.2026.1879274

**Published:** 2026-10-29

**Authors:** Zhongliang Guo, Kei Oyama, Rui Yamaguchi

**Affiliations:** 1 Division of Cancer Systems Biology, Aichi Cancer Center Research Institute, Nagoya, Aichi, Japan; 2 Nagoya University School of Medicine, Nagoya, Aichi, Japan; 3 Division of Cancer Informatics, Nagoya University Graduate School of Medicine, Nagoya, Aichi, Japan

**Keywords:** AlphaFold 3, CDR engineering, conformational ensemble, T-cell receptor, TCR–pMHC

## Abstract

Machine learning-based binding affinity prediction can support T-cell receptor (TCR) engineering, but its reliability remains uncertain when experimental structures of TCRs bound to peptide–major histocompatibility complexes (pMHCs) are unavailable and predicted structures must be used instead. Here, we examined how AlphaFold 3 (AF3) scaffold choice and conformational sampling affect ΔΔG -based prioritization of TCR–pMHC complementarity-determining region (CDR) mutations. Bound–unbound structural comparisons showed that pMHC binding is associated mainly with local CDR remodeling, especially in CDR3 loops, whereas TCR framework regions remain comparatively stable. AF3 generally recovered bound-like TCR conformations, but downstream ΔΔG prediction performance depended strongly on both scaffold source and model architecture. These scaffold-dependent differences indicate that experimental structures remain the most reliable input when available, although such structures are rarely accessible in prospective TCR design settings. Among AF3-based inputs, Prompt-DDG and RDE-PPI were relatively robust to wild-type predicted scaffolds, whereas BA-DDG and H3-DDG benefited more from mutant-specific AF3 scaffolds evaluated by reverse-mutation scoring. Across four templates, AF3-mutant scaffolds gave the highest mean top-10 recovery among the AF3-based inputs for all four predictors. These results support using experimental structures when available and suggest using AF3-mutant scaffolds for recovering top-ranked CDR candidates when only predicted structures are available.

## 1 Introduction

In recent years, machine-learning approaches for protein design have advanced rapidly. Diffusion-based backbone generators such as RFdiffusion (Watson et al., 2023), combined with deep-learning sequence design tools such as ProteinMPNN (Dauparas et al., 2022), have enabled the design of mini-protein binders against diverse targets, and related extensions now support atomic-resolution antibody design (Bennett et al., 2026). In protein structure prediction, the AlphaFold Protein Structure Database has greatly expanded structural coverage of known protein sequence space (Varadi et al., 2022), while AlphaFold 3 has further expanded structure prediction from individual proteins to biomolecular complexes, including protein–protein, protein–nucleic-acid, and protein–ligand assemblies (Abramson et al., 2024). These advances have made it possible to generate hundreds to thousands of candidate binders for a target of interest. However, experimental validation of binding affinity by surface plasmon resonance (SPR) and other biophysical assays remains labor-intensive, requires specialized expertise and expensive equipment, and is difficult to scale to large design libraries (Zhou et al., 2016). Computational methods that prioritize candidates by predicted binding strength are therefore becoming an increasingly important component of protein-design workflows (Guo and Yamaguchi, 2022).

Antibody and T-cell receptor (TCR) engineering are important application areas in which candidate prioritization depends on accurate modeling of protein–protein recognition. Both antibodies and TCRs are heterodimeric immune receptors whose antigen-binding sites are formed by complementarity-determining regions (CDRs). Antibodies comprise paired heavy and light chains, with three CDR loops from each variable domain forming the antigen-binding surface (Sela-Culang et al., 2013). TCRs are typically composed of TCR α and TCR β chains, whose variable domains likewise contribute three CDR loops each; together, these loops recognize antigenic peptides presented by major histocompatibility complex molecules, collectively referred to as peptide–MHC (pMHC) (Rudolph et al., 2006). Among these loops, CDR3 α and CDR3 β are highly sequence-variable and can undergo substantial conformational changes during antigen engagement (McMaster et al., 2024). In both antibody–antigen and TCR–pMHC recognition, local sequence optimization is commonly guided by the predicted mutation-induced change in binding free energy, ΔΔG , relative to a reference complex. By standard convention, negative ΔΔG denotes improved binding relative to the wild type, whereas positive ΔΔG denotes weakened binding. A series of structure-based deep-learning models have been developed for this task, including RDE-PPI (Luo et al., 2023), Prompt-DDG (Wu et al., 2024), BA-DDG (Jiao et al., 2025), and H3-DDG (Xu et al., 2025). These methods use a template complex structure, typically the wild-type structure, together with mutation information to predict ΔΔG , and therefore do not assume that mutant complex structures are experimentally available. Sequence-based affinity predictors provide a fast structure-free alternative (Abbasi et al., 2020; 2021; Li et al., 2021). We include PPAC (Zhang et al., 2025), evaluated on the same mutations, as a reference for whether structure-based models retain an advantage when experimental coordinates are replaced by predicted structures. Structure-based models can also exploit interface geometry, side-chain packing, and conformation-specific information, making it important to test their robustness when experimental structures are replaced by predicted scaffolds. These models are commonly trained and benchmarked on SKEMPI v2 (Jankauskaitė et al., 2019), a manually curated database of experimentally measured mutation effects in protein–protein complexes with known three-dimensional structures. On commonly used SKEMPI v2 splits, reported Pearson correlations between predicted and experimentally measured ΔΔG values often approach 0.7–0.8 (Supplementary Table S1), suggesting potential utility for prioritizing candidate mutants, although performance depends on benchmark design and on the relationship between training and test complexes.

Despite this progress, two assumptions in the prevailing training and evaluation regime limit how confidently current ΔΔG predictors can be deployed at the design stage. First, most benchmarks provide the experimental wild-type complex structure as model input. In many design problems, however, no crystallographic reference structure is available for the relevant complex. Newly characterized targets, engineered variants of poorly studied complexes, and *de novo* binders are often represented only by predicted structures from AlphaFold three or related models. It is therefore unclear whether ΔΔG prediction performance observed on experimental structures transfers to predicted structures. Second, most models score a single static conformation per complex. Biophysical measurements such as SPR, by contrast, report equilibrium binding properties that reflect an ensemble of conformational states. This raises the possibility that single-structure predictions may be sensitive to the particular conformation used as input, because different plausible conformations of the same complex can yield different predicted ΔΔG values. A notable prior example is Deep Local Analysis (DLA) (Mohseni Behbahani et al., 2023), which aggregates predictions over Rosetta backrub-generated conformations (Smith and Kortemme, 2008); however, DLA still uses the experimental wild-type structure as the starting point for ensemble generation.

Here, we examine how predicted structure and ensemble-input choices affect ΔΔG prediction in a biologically motivated setting: TCR–pMHC binding. TCR-based therapeutics have become an active area of cancer immunotherapy. Tebentafusp, a soluble TCR-based bispecific T-cell engager, was approved by the FDA in 2022 (Dhillon, 2022). Because both TCR and antibody recognition depend critically on CDR-mediated binding, CDR loop design represents a shared challenge in TCR and antibody engineering. From the ATLAS database (Borrman et al., 2017), we curated 438 mutation entries with experimentally measured ΔΔG values across 37 unique TCR–pMHC complexes, all associated with resolved three-dimensional structures. Compared with antibody–antigen datasets such as AB-Bind (Sirin et al., 2016), in which antigen size, shape, and molecular architecture vary widely, TCR–pMHC complexes represent a more structurally constrained class of protein–protein interfaces. In these complexes, the TCR engages the peptide–MHC surface through CDR loops from paired TCR α and TCR β variable domains, while the peptide is presented within the conserved MHC peptide-binding groove. This structural regularity reduces confounding variation from antigen size and overall complex architecture, making TCR–pMHC binding a useful testbed for evaluating both ΔΔG prediction under different input structure sources and ensemble-sampling choices and candidate filtering strategies for CDR design.

To systematically evaluate how the structure source and conformational ensemble sampling affect TCR–pMHC ΔΔG prediction, we make four contributions. First, we quantify CDR conformational change upon pMHC engagement across 30 matched bound/unbound TCR pairs from the TCRFlexDock benchmark (Pierce and Weng, 2013) using both C α and common heavy atoms, showing that CDR3 loops and interface side chains exhibit the largest displacements while framework regions remain comparatively stable. Second, we benchmark AlphaFold Server predictions against the same experimental structures at C α , all-heavy-atom, side-chain, interface, and residue-wise resolution. Third, we evaluate four state-of-the-art structure-based ΔΔG predictors—RDE-PPI, Prompt-DDG, BA-DDG, and H3-DDG—on the curated ATLAS mutation set using experimental PDB structures, single AlphaFold-predicted wild-type structures, single AlphaFold-predicted mutant structures, and AlphaFold-predicted conformational ensembles, benchmarking all of them against a sequence-only baseline that receives no structural input. Finally, we quantify prediction uncertainty, evaluate how mutant-ensemble size affects performance, and compare CDR candidate prioritization across four TCR–pMHC templates with at least 20 eligible CDR variants each.

## 2 Results

### 2.1 CDR conformational changes upon pMHC binding

Figure 1A shows a representative bound–unbound TCR pair (PDB 3DXA/3DX9), highlighting the conformational rearrangement associated with pMHC engagement. In the unbound state, the CDR3 loop occupies a position close to the pMHC-binding interface and would sterically clash with the bound pMHC configuration. Upon pMHC binding, this loop is displaced outward and adopts a conformation compatible with peptide recognition. To quantify these binding-associated rearrangements, we computed per-loop C α RMSD between bound and unbound TCR structures across 30 matched pairs (Section 4.1; Figure 1B; Supplementary Table S2). Bound and unbound structures were superposed using framework region C α atoms, allowing loop-specific conformational changes to be evaluated relative to the conserved TCR scaffold.

**FIGURE 1:**
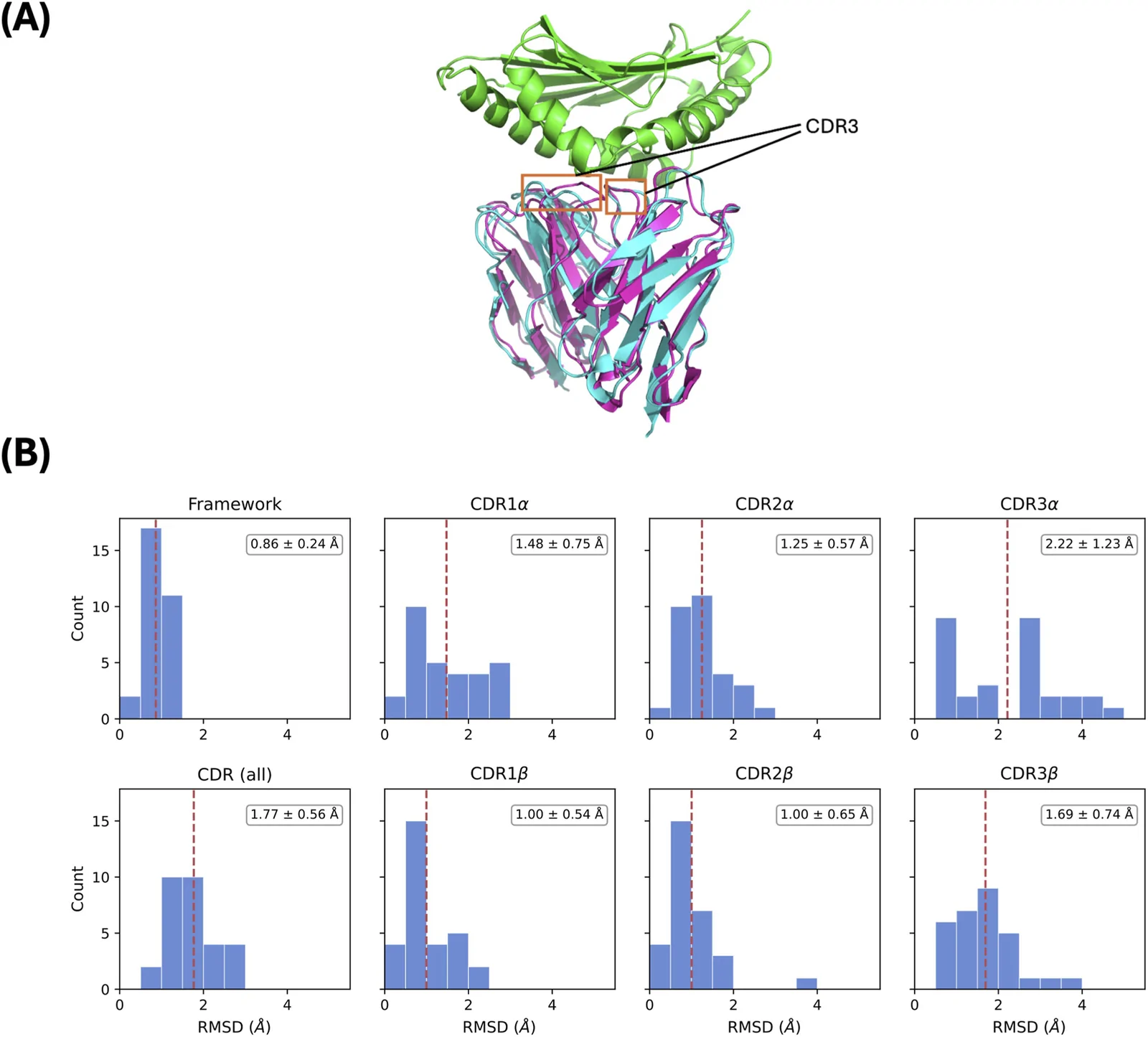
CDR loop conformational changes upon pMHC binding. **(A)** Structural superposition of bound (3DXA) and unbound (3DX9) TCR structures. The pMHC complex is shown in green, the unbound TCR in magenta, and the bound TCR in cyan; the orange boxes highlight the CDR3 region. In the unbound state, the CDR3 loop occupies a position close to the pMHC-binding interface, whereas upon binding it is displaced outward and adopts a conformation compatible with peptide engagement. **(B)** Distribution of C α RMSD values between bound and unbound TCR structures across 30 matched pairs. Top row: framework region and α -chain CDR1–3; bottom row: all CDR positions combined and β -chain CDR1–3. Red dashed lines indicate mean RMSD. The framework region shows consistently low RMSD, whereas CDR loops, especially CDR3 α and CDR3 β , show larger and more variable conformational changes upon pMHC binding.

The framework regions showed low RMSD across the dataset (0.86 ± 0.24 Å), confirming that the TCR immunoglobulin scaffold is largely conserved between bound and unbound states. In contrast, the combined CDR regions showed higher RMSD (1.77 ± 0.56 Å), indicating that pMHC binding is associated primarily with local remodeling of the antigen-recognition loops rather than global rearrangement of the TCR framework region.

Among individual loops, CDR3 showed the largest binding-associated displacements. Mean RMSD values were 2.22 ± 1.23 Å for CDR3 α and 1.69 ± 0.74 Å for CDR3 β , consistent with the central role of CDR3 loops in peptide recognition and antigen specificity (Szeto et al., 2021). CDR1 and CDR2 loops exhibited smaller but detectable changes, with α -chain loops showing greater mobility than their β -chain counterparts: CDR1 α and CDR2 α had mean RMSD values of 1.48 ± 0.75 Å and 1.25 ± 0.57 Å, respectively, whereas CDR1 β and CDR2 β both averaged approximately 1.00 Å.

The broad distribution of CDR3 RMSD values indicates that the extent of conformational rearrangement varies across TCR–pMHC systems (Figure 1B). Together with the representative structural superposition, these results suggest that pMHC recognition can involve substantial CDR loop repositioning rather than simple rigid-body docking of a preformed binding site, supporting the use of ensemble-based approaches that explicitly sample the conformational landscape of CDR loops during binding assessment.

An atom-level analysis confirmed that this pattern also held when all heavy atoms and side-chain atoms were considered. After the same framework C α alignment, pooled all-heavy-atom RMSD averaged 1.22 ± 0.23 Å in the framework and 2.27 ± 0.57 Å across all CDRs; side-chain RMSD increased from 1.48 ± 0.25 Å in the framework to 2.73 ± 0.69 Å across all CDRs. For TCR residues contacting pMHC within 5 Å in the bound reference, side-chain RMSD averaged 3.13 ± 1.00 Å. Residue-wise profiles localized the largest mean displacements to the CDR3 regions, especially CDR3 α (Figure 2), supporting binding-associated local remodeling at the side-chain and interface levels.

**FIGURE 2:**
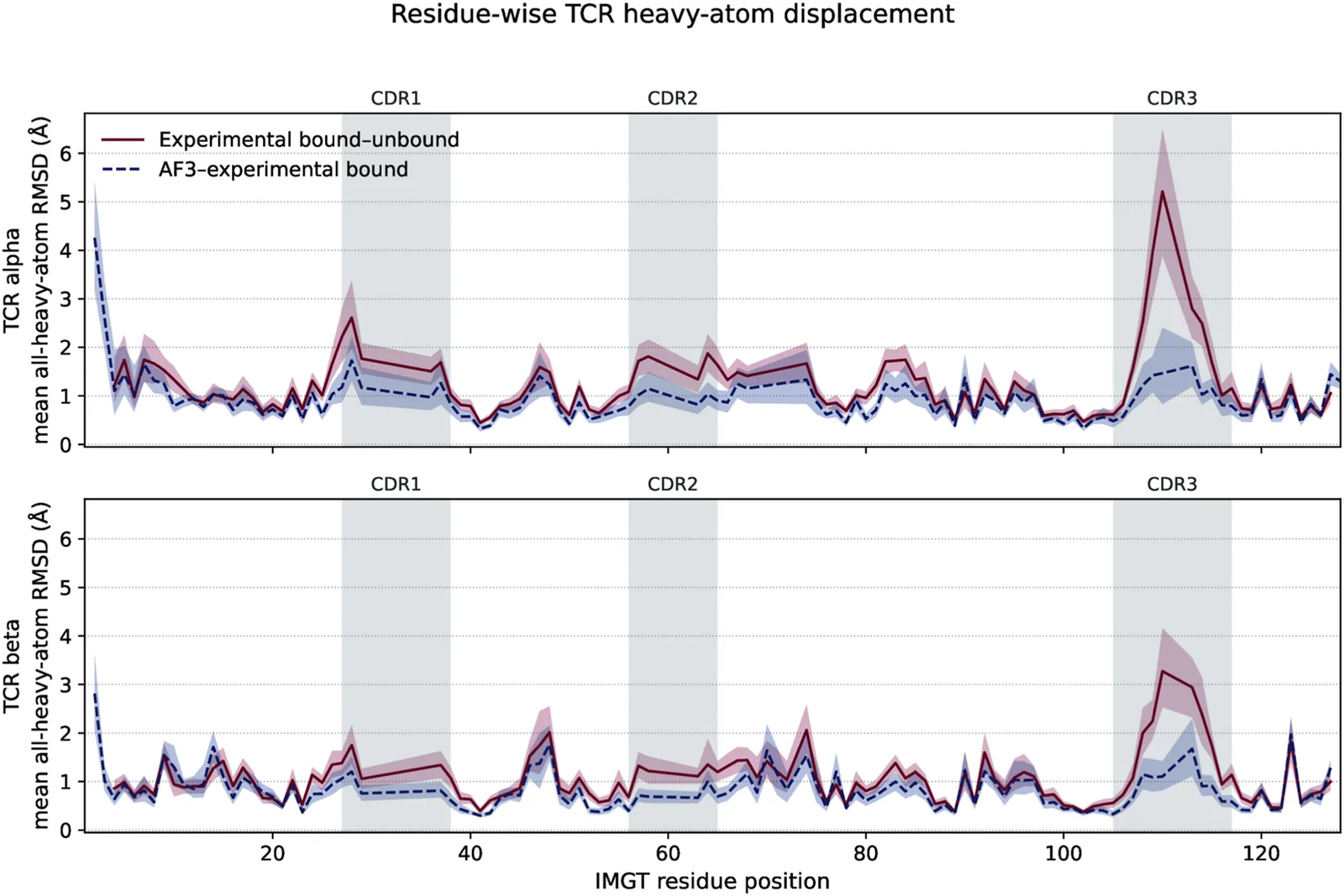
Residue-wise common-heavy-atom RMSD after TCR-framework C α alignment. Profiles show the mean across matched complexes for experimental bound–unbound comparisons and comparisons between top-ranked AF3 predictions and experimental bound structures, separately for the TCR α and β chains; shaded intervals show 10,000-replicate bootstrap 95% CIs, and gray background bands identify IMGT CDR1, CDR2, and CDR3 regions. The largest experimental binding-associated displacements localize to the CDRs, particularly CDR3, while the AF3 profiles are generally lower and more uniform. Exact per-residue values and sample counts are provided in Supplementary Table S5.

### 2.2 AlphaFold server captures bound-like TCR–pMHC complex structures

In many protein design and binding-assessment workflows, experimentally determined structures of designed or candidate binders are unavailable. Predicted complex structures are therefore frequently used as structural inputs for downstream analyses of binding interfaces and interaction quality. AlphaFold Server provides high-accuracy structure prediction through a web interface, making complex-structure prediction available to researchers without a local machine-learning environment (Abramson et al., 2024). We therefore evaluated how accurately AlphaFold Server predicts TCR–pMHC complexes by comparing the top-ranked prediction for each of the 30 complexes with the corresponding experimental bound structure.

Overall, AlphaFold Server reproduced the TCR regions with high accuracy in most cases. The TCR framework regions were closely reproduced, with a mean C α RMSD of 0.78 ± 0.25 Å after superposition on the TCR framework region (Section 4.2; Table 1). This value was comparable to the experimentally observed bound–unbound framework region RMSD of 0.86 ± 0.24 Å, indicating that the conserved TCR scaffold is well captured by AlphaFold Server.

**TABLE 1:** C α RMSD (Å) of AlphaFold Server predictions compared with experimental bound structures, alongside bound–unbound RMSD values for reference. All values in this table were computed after superposition on TCR framework region C α atoms. The Whole complex C α row reports the whole-complex RMSD after the same TCR-framework region superposition and therefore reflects both local modeling accuracy and the placement of pMHC relative to the TCR. No bound–unbound value is reported for this row because the unbound structures contain only the TCR. Statistics are reported across all 30 complexes.

Region	AlphaFold vs. Experimental				Bound vs. Unbound			
	Mean	Std	Min	Max	Mean	Std	Min	Max
CDR1 α	0.96	0.81	0.15	3.32	1.48	0.75	0.32	2.95
CDR2 α	0.68	0.25	0.35	1.34	1.25	0.57	0.47	2.61
CDR3 α	0.95	0.83	0.30	4.62	2.22	1.23	0.68	4.93
CDR1 β	0.53	0.23	0.21	1.27	1.00	0.54	0.28	2.22
CDR2 β	0.53	0.24	0.18	1.23	1.00	0.65	0.25	3.81
CDR3 β	0.72	0.40	0.23	2.05	1.69	0.74	0.86	3.88
Framework region	0.78	0.25	0.51	1.72	0.86	0.24	0.43	1.26
CDR (all)	0.86	0.47	0.35	2.55	1.77	0.56	0.84	2.83
Whole complex C α	1.63	0.85	0.73	4.46	—	—	—	—

Predicted CDR conformations were closer to the experimental bound structures than were the corresponding unbound experimental conformations (Table 1). The combined CDR RMSD for AlphaFold predictions averaged 0.86 ± 0.47 Å, compared with 1.77 ± 0.56 Å for bound–unbound pairs. This difference was especially pronounced for the CDR3 loops, which showed the largest binding-associated rearrangements in the experimental structures: CDR3 α RMSD decreased from 2.22 ± 1.23 Å in bound–unbound comparisons to 0.95 ± 0.83 Å in AlphaFold predictions, and CDR3 β decreased from 1.69 ± 0.74 Å to 0.72 ± 0.40 Å.

These results indicate that AlphaFold Server often predicts bound-like CDR loop conformations with high local accuracy. However, prediction quality was not uniform across complexes (Supplementary Table S3). Because structures were superposed on the TCR framework region, the whole-complex C α RMSD evaluates the placement of pMHC relative to the TCR in addition to local modeling accuracy. This RMSD ranged from 0.73 to 4.46 Å, with a mean of 1.63 ± 0.85 Å (Table 1; Supplementary Table S3). Several outliers therefore showed substantial deviations in TCR–pMHC docking geometry despite accurate local modeling of the TCR framework region and CDR loops. Thus, while AlphaFold Server captures bound-like TCR conformations for many complexes, the relative orientation of TCR and pMHC remains challenging in some systems.

The heavy-atom comparison also showed that the AF3 predictions more closely resembled the experimental bound CDR geometry (Figure 2; Supplementary Tables S4,5). These predictions had all-heavy-atom RMSDs of 1.24 ± 0.26 Å for the framework and 1.28 ± 0.44 Å for the CDRs, while side-chain RMSDs were 1.58 ± 0.30 Å and 1.59 ± 0.47 Å, respectively. Relative to experimental bound–unbound change, AF3 CDR side-chain RMSD was lower in 29 of 30 complexes (paired mean difference, −1.14 Å). At the experimental 5-Å TCR–pMHC interface, AF3 side-chain RMSD averaged 1.75 ± 0.65 Å and was lower in 27 of 30 complexes (paired mean difference, −1.37 Å). Thus, AF3 predictions also more closely resembled the experimental bound structures when side-chain atoms and TCR residues at the experimentally defined TCR–pMHC interface were compared, although the residual 1–2 Å errors remain relevant to geometry-sensitive ΔΔG predictors.

### 2.3 Structure-input choice and model choice both determine ΔΔG prediction accuracy

We sought to identify which predictor–scaffold combinations remain accurate when experimental TCR–pMHC structures are replaced by AF3 models and whether aggregation across AF3 structures improves performance. We therefore evaluated four structure-based predictors across the five structural-input regimes defined in Section 4.3 and compared them with PPAC (Zhang et al., 2025) as a sequence-only reference (Table 2). The evaluated 438-variant cohort and per-template Pearson r , RMSE, and MAE results are provided in Supplementary Tables S6-9.

**TABLE 2:** ΔΔG prediction performance of RDE-PPI, Prompt-DDG, BA-DDG, and H3-DDG on the ATLAS evaluation set under the five structure-input regimes defined in Section 4.3.

#	Structural input	RDE-PPI			Prompt-DDG			BA-DDG			H3-DDG		
		r	RMSE	MAE	r	RMSE	MAE	r	RMSE	MAE	r	RMSE	MAE
1	PDB_template	0.840	1.251	0.808	0.822	1.344	0.797	0.826	1.281	0.812	0.795	1.353	0.828
2	wt_af3_single	0.786	1.448	0.993	0.796	1.448	0.885	0.658	1.779	1.126	0.551	1.992	1.193
3a	wt_af3_multi, mean	0.787	1.444	0.987	0.800	1.438	0.880	0.637	1.812	1.141	0.546	1.995	1.214
3b	wt_af3_multi, median	0.787	1.444	0.989	**0.800**	**1.437**	**0.879**	0.629	1.828	1.148	0.541	2.005	1.217
3c	wt_af3_multi, rank-wt. mean	**0.788**	**1.443**	0.986	0.800	1.438	0.880	0.642	1.800	1.137	0.546	1.994	1.214
4	mutant_af3_single	0.734	1.510	0.985	0.756	1.660	1.280	**0.669**	**1.682**	0.988	0.729	1.685	1.025
5a	mutant_af3_multi, mean	0.746	1.481	0.976	0.760	1.652	1.279	0.666	1.687	**0.977**	**0.731**	**1.661**	**0.999**
5b	mutant_af3_multi, median	0.746	1.478	**0.972**	0.761	1.651	1.278	0.664	1.693	0.980	0.730	1.669	1.003
5c	mutant_af3_multi, rank-wt. mean	0.745	1.481	0.976	0.761	1.650	1.278	0.664	1.691	0.978	0.729	1.670	1.003
*Sequence-only reference baseline (no structural input)*													
S	sequence_only (PPAC)	r=0.504				RMSE =1.949				MAE =1.205			

Regimes three and 5 use AF3 ensembles and are reported with three per-mutation aggregation strategies: arithmetic mean, median, and AF3 ranking-score-weighted mean. Metrics are Pearson correlation (r) , root-mean-square error (RMSE; kcal/mol), and mean absolute error (MAE; kcal/mol). Bold values indicate the best AF3-based result for each model and metric, excluding the experimental PDB_template reference; where 2 cells print the same rounded figure, the bold marks the one that is best in the unrounded value. The 95% CIs for every reported r , RMSE, and MAE are provided in Supplementary Table S10; single-versus-arithmetic-mean-ensemble Pearson comparisons and p -values are provided in Supplementary Table S11. The final block row reports PPAC (Zhang et al., 2025), a sequence-only ΔΔG predictor evaluated on the same 438 mutations. PPAC therefore has one set of performance metrics, independent of the five structure-input regimes, and is reported as a non-structural baseline rather than an additional regime.

Experimental wild-type PDB structures provided the best input regime for all four structure-based models. Each model achieved its highest Pearson correlation with PDB_template: RDE-PPI reached r=0.840 , Prompt-DDG r=0.822 , BA-DDG r=0.826 , and H3-DDG r=0.795 . This result is consistent with the original development setting of these models, in which benchmark training and evaluation are primarily based on experimentally resolved wild-type complex structures from SKEMPI v2, whereas mutant complex structures are generally unavailable. Within this regime, RDE-PPI gave the best Pearson correlation and RMSE, whereas Prompt-DDG gave the lowest MAE.

Replacing experimental structures with AF3-predicted wild-type scaffolds affected the models unevenly. Prompt-DDG was the most robust: its Pearson correlation decreased only modestly, from r=0.822 with experimental wild-type structures to r=0.796 with AF3-WT inputs. RDE-PPI was close behind, retaining r=0.786 under AF3-WT input. These results are consistent with architectures of the two models. Prompt-DDG encodes mutation-centered microenvironments through hierarchical prompts that summarize residue type, angular statistics, and local conformational information, which may make it less sensitive to small global scaffold differences than models that rely directly on backbone-conditioned sequence likelihoods. RDE-PPI, in turn, predicts ΔΔG by modeling side-chain conformational flexibility through estimated rotamer distributions and entropy. This distributional formulation may make the model less dependent on any single predicted side-chain conformation and therefore more tolerant of small coordinate-level errors in AF3-derived scaffolds, partly explaining its relative tolerance to AF3-WT inputs. We examine structural and confidence correlates of these scaffold-dependent errors in Section 2.4.

By contrast, BA-DDG and H3-DDG showed larger performance losses on AF3-WT structures. BA-DDG decreased from r=0.826 with experimental wild-type structures to r=0.658 with AF3-WT scaffolds, and H3-DDG decreased from r=0.795 to r=0.551 . BA-DDG estimates ΔΔG from inverse-folding sequence likelihoods under a thermodynamic cycle that explicitly contrasts bound and unbound states, whereas H3-DDG extends inverse-folding-based modeling with hierarchical many-body attention to capture local and global higher-order interactions. These mechanisms are powerful when the input backbone accurately represents the interaction geometry, but they may also be sensitive to AF3-induced perturbations in local contact topology. This sensitivity may be particularly relevant for TCR–pMHC complexes, where CDR3 loops and the bound peptide often mediate small but functionally important conformational differences.

Both models recovered part of the correlation lost with AF3-WT scaffolds when AF3-mutant scaffolds were evaluated by inversion–negation: BA-DDG increased from r=0.658 to 0.669, and H3-DDG increased from r=0.551 to 0.729. In this workflow, AF3 generates a scaffold from each mutant sequence before the ΔΔG predictor scores the reverse mutant-to-WT substitution and the sign is inverted. These correlations therefore describe the combined AF3 structure-generation and ΔΔG -prediction workflow.

Aggregation across AF3 structures had model- and scaffold-dependent effects (Supplementary Tables S10,11). On AF3-mutant scaffolds, arithmetic-mean aggregation increased Pearson r from 0.734 to 0.746 for RDE-PPI $\left(p=1.31\times 10^{-5}\right)$ and from 0.756 to 0.760 for Prompt-DDG (p=0.0199) . On AF3-WT scaffolds, it increased Pearson r from 0.796 to 0.800 for Prompt-DDG (p=0.00654) , whereas the correlation for BA-DDG decreased from 0.658 to 0.637 $\left(p=7.51\times 10^{-6}\right)$ . The other four single-versus-mean comparisons did not meet the 0.05 threshold. Mean and median aggregation gave similar results, and AF3-ranking-score weighting did not consistently outperform uniform averaging. Complete effect estimates and 95% CIs are reported in Supplementary Tables S10,11.

Global and per-template 95% CIs for prediction accuracy are reported in Supplementary Tables S10,12. Per-template Pearson r CIs could be estimated for 17 of the 37 templates because they contained at least four measured variants. Of these 17 templates, ten had 4–14 variants; the remaining seven had at least 20 variants. Across the four predictors and five structural-input regimes, the median Pearson r 95% CI width was 1.57 for templates with 4–14 variants and 0.48 for templates with at least 20 variants. We therefore use n≥20 as the threshold for cautious interpretation of individual-template results, treat results from smaller cohorts as exploratory, and base comparisons among structural-input regimes on the complete 438-variant cohort.

In the saturation analysis of 100 randomly selected mutations, increasing mutant-specific AF3 ensembles from 20 to 100 structures produced no consistent improvement in Pearson r , RMSE, or MAE across predictors or aggregation rules (Supplementary Figure S1; Supplementary Tables S13,14). Using more than 20 mutant-specific AF3 structures did not improve ΔΔG prediction in this cohort, although additional structures may sample conformations not represented among the first 20.

The sequence-only PPAC reference reached r=0.504 , RMSE =1.949 kcal/mol, and MAE =1.205 kcal/mol on the same 438 mutations (Table 2). All four structure-based predictors had higher point correlations with experimental PDB scaffolds. This advantage remained largest for RDE-PPI and Prompt-DDG on AF3-WT scaffolds and narrowed for BA-DDG and H3-DDG; the corresponding 95% CIs are reported in Supplementary Table S10.

We also examined whether the relative robustness of RDE-PPI and Prompt-DDG on the predicted scaffolds varied with template overlap between the predictors’ training-data source, SKEMPI 2.0, and the ATLAS test set. Fifteen of the 37 ATLAS template IDs also occurred among the SKEMPI template IDs. The shared-PDB-ID group contained 356 ATLAS records across 15 templates, and the no-shared-PDB-ID group contained 82 records across 22 templates. Both predictors had lower point estimates of Pearson r in the no-shared-ID group on experimental WT and AF3-WT single scaffolds (Supplementary Table S21A). When experimental WT structures were replaced by AF3-WT single scaffolds, Prompt-DDG showed a smaller correlation loss than RDE-PPI in the shared-ID group ( Δr=−0.021 versus −0.059 ), whereas RDE-PPI showed a smaller loss in the no-shared-ID group ( −0.027 versus −0.069 ) (Supplementary Table S21B). The 95% CIs for the between-group differences in correlation change included zero for both predictors (Supplementary Table S22). These descriptive comparisons do not isolate the contribution of training overlap to scaffold robustness.

Overall, experimental PDB templates remain the most reliable structural input for TCR–pMHC mutant ΔΔG prediction. Among AF3-based settings, Prompt-DDG was most robust to substitution of experimental structures with AF3-WT scaffolds, whereas BA-DDG and H3-DDG benefited more from AF3-mutant scaffolds evaluated by inversion–negation. Scaffold regime and model architecture had larger and more consistent effects than simple *post hoc* averaging over AF3 samples. We next test whether AF3-WT structural features identify scaffold-dependent errors (Section 2.4) and then examine how the accuracy patterns translate into cross-template candidate ranking (Section Equation 2.5).

### 2.4 Structural correlates of AF3-WT prediction error

To compare prediction accuracy between AF3-WT and the experimental PDB scaffold, we defined the absolute-error change for variant i and model m as

$$\Delta E_{im}=\left|{\hat{\Delta \Delta G}}_{im}^{\;AF3-WT}-\Delta \Delta G_{i}^{\;exp}\right|-\left|{\hat{\Delta \Delta G}}_{im}^{\;PDB}-\Delta \Delta G_{i}^{\;exp}\right|.$$

Positive values indicate greater prediction error with AF3-WT. We examined whether this outcome was associated with TCR–pMHC contact disagreement, CDR3 RMSD, pTM, or ipTM (Figure 3A; Supplementary Tables S15-17). The statistical analysis is described in Section 4.4.

**FIGURE 3:**
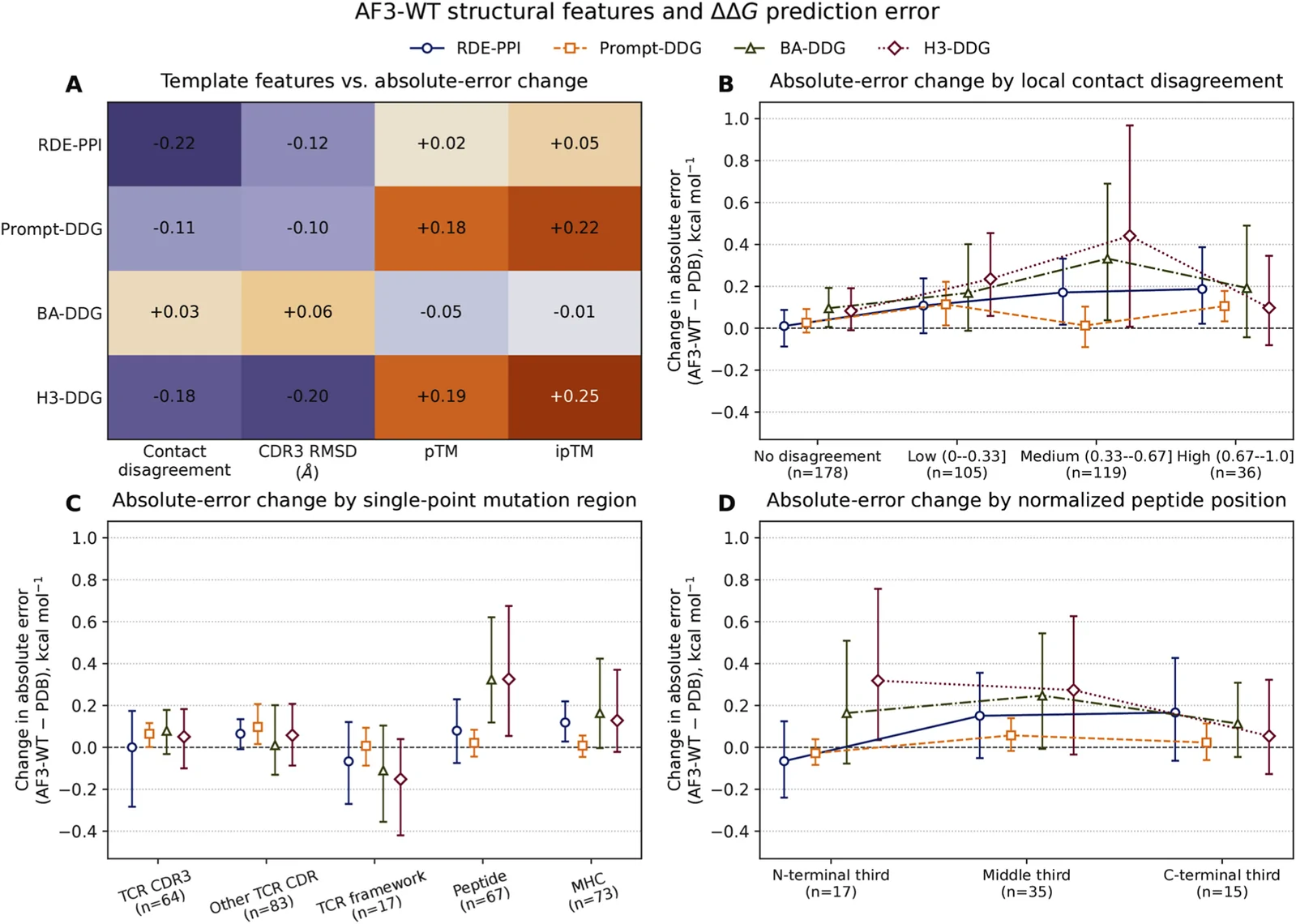
AF3-WT structural features and the change in absolute ΔΔG prediction error relative to the experimental PDB scaffold. The change in absolute prediction error is the AF3-WT absolute error minus the PDB-scaffold absolute error for the same variant, so positive values indicate worse prediction on AF3-WT. **(A)** Spearman ρ across 37 templates; each template feature was paired with the mean absolute-error change across that template’s variants. Contact disagreement is the 5-Å TCR–pMHC contact-set Jaccard distance; CDR3 RMSD was measured after TCR-framework superposition. The cells display the 16 individual predictor–feature associations. The eight additional pairwise-contrast–feature associations are reported in Supplementary Table S17; none of the 24 prespecified associations had an adjusted q<0.05 . **(B)** Absolute-error change by mutation-local contact-disagreement stratum. A mutation-local contact was defined as a TCR–pMHC residue pair involving a mutated residue and containing at least one inter-residue heavy-atom pair within 5 Å. Zero disagreement means that the experimental and AF3-WT structures had the same local residue-contact set, including cases with no local contacts in either structure. **(C)** Absolute-error change by region among 304 single-point mutations. **(D)** Absolute-error change by normalized position among 67 single-point peptide mutations. In B–D, points are means after first averaging variant-level values within each template and category, error bars are 95% template-block bootstrap CIs, and labels give raw variant counts. Connecting lines are descriptive; neither the contact strata nor peptide thirds showed a monotonic error relationship.

None of these global features provided a reliable failure indicator. Figure 3A displays the 16 associations between the four individual predictors and four prespecified features. The eight additional associations for the BA-DDG-minus-Prompt-DDG and H3-DDG-minus-Prompt-DDG absolute-error-change contrasts are reported in Supplementary Table S17. Across all 24 prespecified associations, the Benjamini–Hochberg-adjusted q -values were at least 0.676. For the individual models, contact-disagreement correlations ranged from ρ=−0.220 to 0.029, CDR3-RMSD correlations from −0.200 to 0.057, pTM correlations from −0.052 to 0.186, and ipTM correlations from −0.013 to 0.255. Contact results were similarly weak at 4- and 6-Å sensitivity cutoffs. Exploratory AF3 confidence and local-geometry features likewise showed no pattern that reproduced consistently across predictors (Supplementary Table S17). Global pTM or ipTM thresholds consequently cannot be recommended as a gate for downstream AF3-based ΔΔG scoring in this cohort.

Mutation-local contact disagreement describes how much the TCR–pMHC contacts around the mutated residue(s) differ between the experimental and AF3-WT structures; larger values indicate greater disagreement. This feature showed a more suggestive relationship with the absolute-error change (Figure 3B). Its rank association was ρ=0.445 for BA-DDG and 0.378 for H3-DDG, compared with 0.216 for Prompt-DDG and 0.166 for RDE-PPI, but all adjusted q -values were at least 0.611. Absolute-error changes were also non-monotonic across the contact-disagreement strata. Detailed confidence intervals are provided in Supplementary Table S17. Local contact change is therefore a candidate warning signal for BA-DDG and H3-DDG, not a validated decision rule.

Mutation region gave the clearest descriptive stratification (Figure 3C). Among 304 single-point mutations, the absolute-error change for peptide substitutions was 0.324 kcal/mol for BA-DDG and 0.326 kcal/mol for H3-DDG, compared with 0.022 kcal/mol for Prompt-DDG and 0.080 kcal/mol for RDE-PPI. This pattern did not resolve to a particular peptide location: all adjusted q -values were at least 0.785, and the absolute-error change did not consistently increase or decrease across the N-terminal, middle, and C-terminal thirds (Figure 3D). Detailed confidence intervals are provided in Supplementary Table S17. Thus, the present data support a region-level caution for BA-DDG and H3-DDG on AF3-WT peptide substitutions, while offering no evidence for a specific vulnerable peptide position.

### 2.5 Candidate-prioritization performance depends on structure input, model, and template

The global metrics in Section 2.3 assess agreement with experimental ΔΔG across all mutants, whereas candidate prioritization asks whether stabilizing CDR variants are placed near the top of a template-specific ranking. We evaluated this task across four ATLAS templates, each with at least 20 measured variants carrying at least one CDR mutation, for a combined cohort of 191 variants. The 20-variant cutoff follows the uncertainty analysis in Section 2.3, which showed narrower per-template Pearson r confidence intervals for templates with at least 20 variants than for templates below this cutoff. For every template, model, and structural-input setting, we calculated Spearman ρ and the number of the ten most-stabilizing experimental variants recovered in the predicted top 10. Table 3 reports the median per-template correlation and mean top-10 recovery across the four templates; Supplementary Figure S2 shows the correlation distributions, and Supplementary Table S18 provides the complete per-template results.

**TABLE 3:** Candidate prioritization across four ATLAS templates, each with at least 20 measured variants carrying at least one CDR mutation (1AO7, 1MI5, 2BNR, and 3QDJ; combined n=191 ).

Structural input	RDE-PPI	Prompt-DDG	BA-DDG	H3-DDG
PDB_template	0.854 (8.5)	0.820 (8.3)	0.607 (7.3)	0.558 (7.0)
wt_af3_single	**0.712** (6.5)	**0.789** (7.3)	0.529 (5.0)	0.383 (3.3)
wt_af3_multi, mean	0.707 (6.3)	0.787 (6.8)	0.530 (4.5)	0.389 (3.5)
mutant_af3_single	0.605 (7.0)	0.682 (**7.5**)	**0.617** (**7.3**)	0.687 (**7.3**)
mutant_af3_multi, mean	0.617 (**7.3**)	0.683 (**7.5**)	0.608 (**7.3**)	**0.691** (**7.3**)

Each cell reports the median per-template Spearman ρ ; parentheses show the mean number of the ten most-stabilizing experimental variants recovered per template. The _multi settings use arithmetic-mean aggregation across AF3 samples. Bold values indicate the best AF3-based median correlation and mean recovery for each model, with ties retained. Complete per-template correlations, 95% CIs, and top- K results are provided in Supplementary Table S18, and the correlation distributions are shown in Supplementary Figure S2.

Experimental TCR–pMHC structures remain the first-choice input when available. With the experimental scaffold, mean top-10 recovery was 8.5 for RDE-PPI, 8.3 for Prompt-DDG, 7.3 for BA-DDG, and 7.0 for H3-DDG (Table 3). Experimental structures therefore gave the highest mean top-10 recovery for RDE-PPI and Prompt-DDG, tied for the highest recovery for BA-DDG, and were close to the highest recovery for H3-DDG (7.0 versus 7.3). These results support using an experimental complex whenever one is available.

When no experimental structure was available, AF3-mutant scaffolds provided the highest mean top-10 recovery across the four evaluated templates for all predictors. For RDE-PPI, Prompt-DDG, BA-DDG, and H3-DDG, respectively, the best AF3-mutant setting recovered a mean of 7.3, 7.5, 7.3, and 7.3 of the top 10 candidates, compared with 6.5, 7.3, 5.0, and 3.5 for the best AF3-WT setting. AF3-mutant scaffolds can therefore be useful when top-candidate recovery is the primary objective.

Spearman correlations remained model- and template-dependent (Supplementary Figure S2). Prompt-DDG with a single AF3-WT scaffold had the highest AF3-based median correlation (ρ=0.789) , with per-template values ranging from 0.697 to 0.914. For this four-template cohort, the correlation and recovery metrics address complementary objectives: AF3-WT with Prompt-DDG gave the strongest overall rank agreement, whereas AF3-mutant scaffolds provided the more consistent choice across predictors for recovering the top candidates.

The 1AO7 cohort, highlighted in Supplementary Figure S2, provides one example of this dependence (Table 4; SupplementaryTables S19,20). With the experimental scaffold, all four models achieved ρ=0.898 –0.930 and recovered nine of the top 10 candidates. With AF3-WT, Prompt-DDG retained ρ=0.914 –0.915 and recovered 4–5 candidates, whereas BA-DDG and H3-DDG had ρ=0.455 –0.476 and recovered none. In contrast, AF3-mutant inputs yielded ρ=0.836 –0.896 and recovered 8–9 candidates across all four models. This 1AO7 example shows how AF3-mutant inputs can retain strong top-candidate recovery across predictor architectures.

**TABLE 4:** Candidate prioritization on the n=126 1AO7 ATLAS variants carrying at least one CDR mutation.

Structural input	RDE-PPI	Prompt-DDG	BA-DDG	H3-DDG
PDB_template	0.920 (9)	0.930 (9)	0.907 (9)	0.898 (9)
wt_af3_single	0.820 (3)	0.914 (5)	0.458 (0)	0.470 (0)
wt_af3_multi, mean	0.809 (3)	**0.915** (4)	0.455 (0)	0.476 (0)
mutant_af3_single	0.857 (**8**)	0.839 (**8**)	0.880 (**9**)	0.884 (**9**)
mutant_af3_multi, mean	**0.857** (**8**)	0.836 (**8**)	**0.896** (**9**)	**0.896** (**9**)

Each cell reports Spearman ρ between predicted and experimental ΔΔG ; parentheses show the number of the ten most-stabilizing experimental CDR mutants recovered in the model’s top-10 predicted list. The _multi settings use arithmetic-mean aggregation across AF3 samples. Bold values indicate the best AF3-based result for each model; where 2 cells print the same rounded ρ , the bold marks the one with the higher unrounded value. Full top- K precision and per-mutation rankings are provided in Supplementary Tables S19,20.

Overall, an experimental TCR–pMHC structure should be used when available. Results from these four templates suggest using AF3-mutant scaffolds for ΔΔG -based top-candidate selection when experimental structures are unavailable. A single AF3-WT scaffold remains useful with Prompt-DDG when overall rank correlation is the primary metric. Because ranking performance varied by model and template, the selected workflow should be validated for the target TCR–pMHC system before it is used to guide experiments.

## 3 Discussion

This study provides practical guidance for applying structure-based ΔΔG predictors with experimental and AF3-derived TCR–pMHC scaffolds. Experimental wild-type structures gave the strongest overall performance, and their advantage over the sequence-only PPAC reference demonstrates the value of structural information when the structure and predictor are well matched. In the complete 438-variant analysis, Prompt-DDG and RDE-PPI retained relatively strong global accuracy on AF3-WT, whereas mutant-specific scaffolds improved the AF3-based performance of BA-DDG and H3-DDG. In the candidate-prioritization analysis of four templates with at least 20 eligible CDR variants, the best AF3-mutant setting achieved higher mean top-10 recovery than the best AF3-WT setting for every predictor. AF3-WT with Prompt-DDG was useful for overall ranking and had the highest median Spearman correlation among the AF3-based settings. Together, these results support using experimental structures when available and suggest using AF3-mutant scaffolds for top-candidate selection when experimental structures are unavailable.

The AF3-mutant regime evaluates a workflow that combines AF3 structure generation with ΔΔG prediction, rather than the ΔΔG predictor alone. With an experimental or AF3-WT scaffold, the predictor scores the forward wild-type-to-mutant substitution on a WT structural background. With an AF3-mutant scaffold, AF3 first generates a structure from the mutant sequence and may therefore encode mutation-associated structural differences before the predictor scores the reverse mutant-to-WT substitution and the sign is inverted. The ΔΔG predictor therefore no longer evaluates the substitution only on a fixed WT structural background; it may also use mutation-associated structural information already present in the AF3-mutant scaffold. In this sense, part of the predictive task may shift from the ΔΔG predictor to AF3. Because both the scaffold and scoring direction change, the reported performance reflects their combined effect. Accordingly, the four-template results suggest using the complete AF3-mutant workflow for top-candidate selection when experimental structures are unavailable.

Whether AF3-mutant scaffolds reproduce true mutant conformations cannot be determined without paired experimental mutant structures, which were unavailable for this cohort. A mutant generated by side-chain modeling on an experimental WT structure could be used to compare the resulting ΔΔG prediction accuracy with that obtained from an AF3-mutant scaffold. However, without an experimental mutant structure, this comparison could not determine which model more closely reproduces the true mutant conformation. It would also address a different use case because it requires an experimental WT structure, whereas AF3-mutant scaffolds can be generated when only protein sequences are available. As an independent assessment of AF3 structure prediction, we compared sequence-based AF3 models with 30 experimental bound TCR–pMHC structures. AF3 reproduced bound-like CDR geometry, with a mean combined-CDR C α RMSD of 0.86 Å and a mean CDR side-chain RMSD of 1.59 Å. Although this benchmark does not test structural changes caused by individual mutations, it provides evidence that AF3 can generate bound-like TCR/CDR geometry from sequence.

The structural error-feature analysis further clarifies how measured AF3 structural differences relate to changes in ΔΔG prediction error. Template-level contact disagreement, CDR3 RMSD, pTM, and ipTM were not reliably associated with absolute-error changes, and the broader confidence panel yielded no predictor-independent failure signature. High global AF3 confidence is therefore not sufficient to guarantee robust downstream ΔΔG prediction. Mutation-local contact disagreement was suggestive for BA-DDG and H3-DDG but did not support a stable threshold, while AF3-WT peptide substitutions showed the largest absolute-error changes for these two predictors. These findings justify extra caution and cross-model validation for peptide mutations, while treating pTM, ipTM, RMSD, peptide position, and contact disagreement as descriptive features rather than validated screening thresholds.

The atom-level analysis supplies complementary structural context. Bound–unbound changes remained concentrated in CDR and interface side chains, while the AF3 predictions were closer to the experimental bound geometry than the corresponding unbound TCR for CDR side chains in 29 of 30 complexes. The residual AF3 interface side-chain RMSD averaged 1.75 Å, a scale at which contacts, hydrogen-bond geometry, and rotamer packing can change. Consequently, residual local side-chain deviations in AF3 models may perturb the interaction geometry used by ΔΔG predictors, providing a plausible structural explanation for their generally higher accuracy with experimental scaffolds.

Simple AF3 aggregation did not resolve this limitation. At the 0.05 Williams-test threshold, three of eight single-versus-mean Pearson comparisons improved, one deteriorated, and four did not differ; increasing mutant ensembles from 20 to 100 structures likewise produced no consistent performance gain in the fixed saturation cohort. These findings concern downstream metric saturation, not conformational coverage. AF3 samples are stochastic, sequence-conditioned structural hypotheses and are not weighted draws from a Boltzmann equilibrium distribution. Mean, median, or AF3-ranking-score aggregation therefore has no necessary thermodynamic interpretation, and the lack of improvement should not be read as evidence that conformational dynamics are unimportant.

Indeed, experimental and simulation studies show why static structural similarity can be incomplete. Ma et al. showed that neoantigen discrimination can emerge from dynamic and allosteric processes within the peptide–MHC groove, highlighting how conformational fluctuations can shape TCR recognition despite similar static structures (Ma et al., 2025). Nurzia et al. similarly showed that changes in pMHC dynamics may alter TCR-recognition modes despite minimal differences in experimentally resolved conformations (Nurzia et al., 2012). Rollins et al. used molecular dynamics in a workflow for identifying and ranking neoantigen-specific TCRs (Rollins et al., 2024), whereas Frezzini and Narzi combined molecular dynamics and residue-correlation analyses to investigate the determinants of affinity enhancement in an engineered TCR (Frezzini and Narzi, 2026). These physics-based approaches and AF3 sampling should be viewed as complementary: AF3 offers rapid structural hypotheses at scale, whereas molecular dynamics can probe time-ordered fluctuations, solvent-mediated rearrangements, and ensemble weights under an explicit physical model.

Related structure-informed work also addresses a different but adjacent design question. Ghoreyshi et al. combined alanine-scan data with the coarse-grained RACER-m energy model, multi-template structural modeling, compensatory TCR screening, and AF3 validation to study escape and rescue mutations in the single 1E6–ALW/HLA-A*02:01 system (Ghoreyshi et al., 2026). Our contribution is complementary: we benchmark four structure-based ΔΔG predictors plus a sequence-only baseline across 438 mutations and multiple scaffold regimes, and test cross-template candidate prioritization. Their focused mechanistic framework can propose escape-specific compensatory sequences, whereas our benchmark asks how reliably existing predictors transfer from experimental to AF3-derived scaffolds across a heterogeneous dataset.

Several limitations and future directions should be noted. The 438 measured variants are distributed unevenly across 37 templates; 29 templates contain fewer than ten variants and 20 contain fewer than four, so many template-specific correlations remain imprecise. Restricting candidate prioritization to templates with at least 20 eligible CDR variants supports more precise within-template estimates, but the resulting four-system analysis provides preliminary guidance. Validation in additional independent TCR–pMHC systems is needed to establish broader applicability. The present benchmark used AF3 as the source of predicted conformations and evaluated four structure-based ΔΔG models. Further work with paired experimental mutant structures, larger and more balanced TCR–pMHC datasets, additional predictors, and other structure-generation or conformational-sampling approaches will be valuable to determine how broadly the present findings extend. Candidate approaches include diffusion-based conformational samplers (Lewis et al., 2025), AlphaFold-based flow-matching ensemble generators (Jing et al., 2024), and molecular-dynamics-based ensembles. Within this scope, our results support a practical workflow in which experimental structures are preferred when available, whereas AF3-mutant scaffolds can provide a useful fallback for recovering top-ranked CDR candidates when experimental scaffolds are unavailable.

## 4 Materials and methods

### 4.1 Structural comparison of bound and unbound TCR conformations

To quantify CDR loop conformational changes upon pMHC binding, we analyzed 30 matched pairs of bound and unbound TCR structures from the TCRFlexDock benchmark (Pierce and Weng, 2013). Each pair consists of (i) a TCR extracted from a co-crystal TCR–pMHC complex structure and (ii) the same TCR solved independently in the absence of pMHC. The dataset spans 25 Class I and 5 Class II MHC-restricted complexes.

Residue numbering was assigned using the IMGT scheme (Lefranc et al., 2003) via the ANARCI tool (Dunbar and Deane, 2016), rather than relying on deposited residue numbers, which are inconsistent across PDB entries. CDR loop boundaries were defined in IMGT numbering as CDR1 (positions 27–38), CDR2 (positions 56–65), and CDR3 (positions 105–117). Framework region residues were defined as all non-CDR positions in the TCR variable domains.

For each pair, the unbound TCR structure was superposed onto the bound TCR using singular value decomposition (SVD) applied to framework region C α atoms shared between the two structures. After superposition, the root-mean-square deviation (RMSD) of C α atoms was computed separately for each of the six CDR loops (CDR1–3 on both α and β chains) as well as for the framework region. Structure parsing and superposition were performed with Biopython (Cock et al., 2009).

For the heavy-atom analysis, both experimental bound–unbound pairs and top-ranked AF3–experimental pairs were aligned on framework C α atoms using the same IMGT definitions. Comparisons retained only common IMGT positions with identical residue types and atom names present in both structures. RMSD was pooled over all common heavy atoms or over side-chain atoms after excluding N, C α , C, and O. Reference interface residues were TCR residues with any heavy atom within 5 Å of pMHC in the experimental bound/reference structure. Supplementary Table S4 contains per-complex regional and interface results, while Supplementary Table S5 contains per-residue results. Residue-wise profiles first pooled insertion-coded residues at each base IMGT position within a complex, then plotted the across-complex mean and 10,000-replicate bootstrap 95% CI for positions represented in at least 15 complexes.

### 4.2 AlphaFold server structure prediction of TCR–pMHC complexes

To evaluate the accuracy of state-of-the-art structure prediction for TCR–pMHC complexes, we submitted the same 30 benchmark structures to AlphaFold Server (Abramson et al., 2024). For each complex, input sequences were extracted from the experimental bound structure and truncated to the binding-relevant domains: TCR variable domains (identified via ANARCI IMGT numbering), MHC peptide-binding platform (residues 1–180 for Class I heavy chains; residues 1–95 for Class II α / β chains), and the antigenic peptide. Beta-2-microglobulin was excluded from Class I submissions for consistency across structures. Peptide chains were submitted with structural templates disabled (useStructureTemplate: false), and all other chains were assigned a maxTemplateDate set to the last day of the month preceding each structure’s RCSB PDB release date, ensuring that the target structure itself could not serve as a template.

AlphaFold Server produced five ranked models per complex. Each predicted model was compared against the corresponding experimental structure after superposition on TCR variable-domain framework region C α atoms (as described in Section 4.1). Per-CDR RMSD, framework region RMSD, and whole-complex C α RMSD were all computed under this same TCR-framework region alignment, so the whole-complex value reflects both local modeling accuracy on the TCR and the placement of pMHC relative to the TCR (see Table 1 caption).

The main and heavy-atom summaries use the top-ranked AlphaFold Server output for every complex; models were not selected retrospectively by experimental RMSD. For the heavy-atom cohort comparison, paired mean AF3-minus-bound–unbound differences were assigned percentile 95% CIs by resampling the 30 matched complexes 10,000 times.

### 4.3 ΔΔG prediction across models and structure-input regimes

The evaluation cohort was derived from ATLAS (Borrman et al., 2017) through sequential quality-control filtering. We first corrected 24 corrupted ΔΔG entries in the deposited ATLAS.tsv, where an upstream Unicode round-trip had replaced the ± and − characters with “?”, by recovering the original values from the authors’ source table on their GitHub repository. We also applied the author-supplied GitHub corrections, comprising three row removals and 31 cell replacements. Restricting the corrected table to entries with both measured ΔΔG values and deposited template PDB structures yielded 447 candidate mutants. We then excluded seven entries whose deposited mutant peptide sequence could not be reconciled with the sequence generated by applying PEP_mut to the corresponding wild-type peptide: ATLAS indices 104, 105, 108, and 109 for template 3MV7; 347 for 1AO7; and 690 and 696 for 3QIB. This left 440 mutants. Finally, two entries referencing PDB 1FYT were removed because Biopython’s internal-coordinate builder failed on atoms with undefined occupancy in that structure. The final cohort therefore contained 438 mutants spanning 37 unique TCR–pMHC templates, with mutations on TCR, MHC, and peptide chains (Supplementary Table S6).

To evaluate how model architecture and structural input choice jointly affect mutational ΔΔG prediction for TCR–pMHC complexes, we scored the 438-mutant cohort using four state-of-the-art predictors under five structure-input regimes and one sequence-only predictor that receives no structural input. The structure-based models were trained or evaluated using a common protocol: 3-fold cross-validation on SKEMPI v2 (Jankauskaitė et al., 2019), with folds split by complex to prevent structural overlap between training and validation sets. For ATLAS, predictions are reported throughout as cross-validation-averaged ΔΔG values.

The four predictors were chosen as state-of-the-art structure-based ΔΔG models on SKEMPI v2 (Supplementary Table S1) that are based on different design ideas. RDE-PPI (Luo et al., 2023) uses a flow-based rotamer density prior to model side-chain conformational flexibility and was evaluated using the authors’ released three-fold checkpoint (DDG_RDE_Network_30k.pt); its symmetric wild-type–mutant training objective makes reversed mutation scoring relatively well matched to the model design. H3-DDG (Xu et al., 2025) and BA-DDG (Jiao et al., 2025) are ProteinMPNN-based inverse-folding models that estimate ΔΔG through a thermodynamic-cycle scoring path and use a single-direction MSE objective; H3-DDG adds hypergraph-based hierarchical attention and was trained from scratch on our SKEMPI v2 folds, whereas BA-DDG was used from the authors’ released 3-fold checkpoint fine-tuned on the soluble ProteinMPNN v_48_020 backbone. Prompt-DDG (Wu et al., 2024) instead encodes mutation-centered microenvironments through a hierarchical VQ-VAE prompt codebook and an attention-based ΔΔG prediction; because the released model included the supervised readout but not the unsupervised codebook, we pretrained the codebook from scratch using the published recipe and kept the released readout weights fixed. Apart from these model-specific checkpoints or pretraining steps, entry construction, mutation remapping, structure parsing, inference, and ensemble aggregation were shared across all four models.

PPAC (Zhang et al., 2025) served as the sequence-only reference. It embeds a complex with ESM-2 (Lin et al., 2023) (esm2_t36_3B_UR50D), mean-pooling the layer-33 token representations, and predicts ΔΔG from the difference between the mutant and wild-type embeddings with a multilayer perceptron. Wild-type and mutant sequences were taken from the same AF3 input JSON files that define the structural regimes, so the five models score identical mutations; each complex was presented as two concatenated sides, pMHC (chains A, B, C) followed by TCR (chains D, E), in sorted chain order. Only one checkpoint is released (a single cross-validation fold), so no fold averaging was applied. Because PPAC consumes no coordinates, it yields one prediction per mutation that is identical across all five structure-input regimes.

We compared five structure-input regimes that differ in whether the scaffold is experimental or AlphaFold 3 (AF3)-predicted, whether it represents the wild-type or mutant sequence, and whether a single conformation or an ensemble is used:

1. PDB_template: the forward wild-type → mutant mutation is scored on the experimental wild-type complex (Borrman et al., 2017), matching the SKEMPI v2 evaluation setting.
2. wt_af3_single: the forward mutation is scored on a single AF3 wild-type prediction (seed 1, sample 0).
3. wt_af3_multi: the forward mutation is scored across 100 AF3 wild-type samples per template (10 seeds × 10 samples).
4. mutant_af3_single: a single AF3 mutant prediction (seed 1, sample 0) is used as the scaffold. Because the scaffold already carries the mutant side chain, the mutation is scored in the reverse direction, mutant → wild-type, and the predicted ΔΔG is negated to recover the wild-type → mutant direction; we refer to this reverse-scoring-and-negation procedure as *inversion–negation.*
5. mutant_af3_multi: the same reversed-and-negated scoring procedure is applied to 20 AF3 mutant samples per ATLAS row (2 seeds × 10 samples).

All AF3 predictions used in regimes (2)–(5) were generated on a local AlphaFold three installation rather than the public AlphaFold Server, which limits each user to 30 submissions per day and is therefore incompatible with the dataset sizes used here. For wild-type scaffolds, 100 samples per template (regime 3) were generated to assess the effect of multi-conformation input on ΔΔG prediction with a reasonable ensemble size. For mutant scaffolds, the per-row budget was reduced from 100 to 20 samples (regime 5) to keep the total compute tractable.

For ensemble regimes, per-sample ΔΔG predictions were reduced to one prediction per mutation using three aggregators: arithmetic mean, median, and AF3-ranking-score-weighted mean, with weights shifted to be strictly positive. Predictions were compared with experimental ΔΔG using Pearson correlation, root-mean-square error (RMSE), and mean absolute error (MAE). Uncertainty in all three metrics was quantified using percentile 95% CIs from 10,000 nonparametric bootstrap resamples of the 438 mutation rows (Efron and Tibshirani, 1985). For each model and scaffold type (AF3-WT or AF3-mutant), the Pearson correlation from the single structure was compared with that from arithmetic-mean ensemble aggregation using a two-sided Williams test for dependent correlations that share the same experimental outcome (Steiger, 1980). Paired-bootstrap CIs for Δr were calculated by differencing the single and ensemble correlation within each resample.

To quantify per-template uncertainty, Pearson CIs were calculated separately within each template, model, and structure-input regime using 10,000 within-template bootstrap resamples. CIs were estimated only when a template contained at least four mutations; templates with fewer observations were retained with status not_estimable_n_lt_4. The n≥4 criterion was used only to determine whether an interval could be estimated. For interpretation, n≥20 was used as a threshold for cautious template-level assessment, while estimates from smaller cohorts were retained as exploratory results.

For the mutant-ensemble saturation analysis, 100 mutations were sampled without replacement from the 438-row cohort. For each mutation, 100 structures were generated with local AF3 (10 model seeds × 10 diffusion samples), retaining the original sequence construction, shared-WT-MSA/mutant-query patching, template handling, peptide treatment, mutation remapping, and inversion–negation workflow. Nested ensembles of N=1 , 10, 20, 50, and 100 were evaluated for all four predictors using mean, median, and AF3-ranking-score-weighted aggregation. Ranking weights were $w_{j}=r_{j}-\min_{k}\left(r_{k}\right)+10^{-3}$ and were normalized within each ensemble. Pearson r , RMSE, MAE, and paired differences from the corresponding N=100 result were assigned 95% CIs using 10,000 paired bootstrap resamples of the same 100 mutations. Supplementary Tables S13,14 report the complete metrics and randomly selected cohort.

For the template-overlap analysis, we grouped ATLAS records according to whether their four-character template PDB ID was present in SKEMPI 2.0. We compared RDE-PPI and Prompt-DDG on experimental WT and AF3-WT single scaffolds using pooled Pearson r , RMSE, and MAE, with scaffold changes defined as AF3-WT single minus experimental WT performance on the same records. Percentile 95% CIs were obtained from 10,000 bootstrap resamples. Between-group contrasts were defined as the change in the no-shared-ID group minus that in the shared-ID group (Supplementary Tables S21,22). This grouping identifies overlap with SKEMPI 2.0 by PDB ID; it does not verify training membership in individual checkpoints.

### 4.4 AF3-WT structural error-feature analysis

The error-feature analysis used the common 438-mutation, 37-template cohort. For each predictor, predictions were taken from the model-specific PDB_template and wt_af3_single result files. The wt_af3_single results used the WT AF3 model from seed 1, sample 0. Experimental PDB and AF3-WT residues were aligned separately within chains A–E by sequence, retaining experimental author residue identifiers as canonical keys. TCR–pMHC contacts were defined as residue pairs containing at least one heavy-atom pair within 5 Å, with TCR chains D/E on one side and pMHC chains A/B/C on the other. Contact disagreement was the Jaccard distance, $1-|C_{\text{PDB}}\cap C_{\text{AF}3}|/|C_{\text{PDB}}\cup C_{\text{AF}3}|$ , calculated both over the entire interface and over contacts involving a mutated residue; 4- and 6-Å definitions were evaluated as sensitivity analyses. AF3-WT was fitted to the experimental structure using C α atoms from the ANARCI/IMGT-defined TCR framework, after which pooled CDR3 α/β RMSD and whole TCR–pMHC C α RMSD were measured.

From AF3 summary confidence files we extracted pTM, ipTM, ranking score, fraction disordered, has_clash, and chain-pair ipTM averaged over TCR–pMHC chain pairs with weights proportional to experimental interface-contact counts. From detailed confidence files and model C α B-factors we extracted mean symmetrized PAE and contact probability over mapped experimental interface contacts, CDR3 and peptide pLDDT, and mutation-site pLDDT. Single-point mutations were classified as TCR CDR3, other TCR CDR, TCR framework, peptide, or MHC; peptide positions were normalized from 0 at the N terminus to one at the C terminus. Supplementary Tables S15,16 contain the resulting template- and mutation-level features, respectively.

For variant i and model m , the primary outcome was the change in absolute prediction error after replacing the experimental PDB scaffold with AF3-WT:

$$\Delta E_{im}=\left|{\hat{\Delta \Delta G}}_{im}^{\;AF3-WT}-\Delta \Delta G_{i}^{\;exp}\right|-\left|{\hat{\Delta \Delta G}}_{im}^{\;PDB}-\Delta \Delta G_{i}^{\;exp}\right|,$$

where positive values indicate that substituting AF3-WT for the experimental scaffold increased absolute error. We also analyzed pairwise differences in absolute-error change between BA-DDG and Prompt-DDG and between H3-DDG and Prompt-DDG. Spearman rank correlation was used because the features have heterogeneous, potentially non-normal distributions and the analysis sought monotonic rather than strictly linear associations. Template-level analyses treated each template as one equally weighted observation, using the mean outcome across its variants. Mutation-level analyses ranked the feature and outcome within each template and combined these within-template rank relationships with equal template weights, preventing templates with more variants or different baseline error levels from dominating the results.

Percentile 95% CIs were obtained from 1,000 template-block bootstrap resamples. For two-sided template-level permutation tests, outcomes were shuffled among templates to generate the null distribution. For mutation-level tests, the direction of each template’s within-template rank association was randomly reversed to generate the null distribution while preserving the variants grouped within each template. Benjamini–Hochberg adjustment was applied separately to the prespecified template feature family (contact disagreement, CDR3 RMSD, pTM, and ipTM), the mutation-local contact family, and normalized peptide-position family. Exploratory features were reported with bootstrap CIs but without multiplicity-adjusted tests. For descriptive panels, variant-level values were first averaged within each template and category and then across templates; the bootstrap resampled templates. Contact disagreement was divided into fixed strata (zero, (0,1/3] , (1/3,2/3] , and (2/3,1]) , while peptide position was divided into thirds. Full estimates, CIs, multiplicity-adjusted results, category summaries, and 4/6-Å sensitivity analyses are reported in Supplementary Table S17.

### 4.5 Candidate-prioritization analysis across templates and for 1AO7

For the candidate-prioritization analysis in Section Equation 2.5, the 438-variant evaluation set was grouped by ATLAS template and restricted within each template to variants carrying at least one CDR mutation. CDR-loop residues were identified using the IMGT/ANARCI conventions of Section 4.1. A variant was included if any of its constituent point mutations mapped to a CDR-loop residue, thereby retaining variants with multiple substitutions whenever at least one substituted position satisfied the criterion. Following the n≥20 threshold used for cautious template-level interpretation in Section 4.3, we retained templates with at least 20 eligible variants after this filter. Four templates met this criterion (1AO7, 1MI5, 2BNR, and 3QDJ) and together contained n=191 eligible variants, 191/438≈44% of the cleaned benchmark.

For every eligible template, predictor, and structural-input regime, Spearman ρ between predicted and experimental ΔΔG was computed together with top- K recovery, defined as the number of the K most-stabilizing experimental variants recovered in the predicted top- K list ( K=3 , 5, and 10); top- K precision was the recovery count divided by K . Spearman 95% CIs were calculated separately within each template, predictor, and regime using 10,000 within-template bootstrap resamples, following the procedure described in Section 4.3 (Supplementary Table S18). For each predictor–regime combination, the median, IQR, minimum, and maximum correlation were calculated across the four templates, and the mean of the four top-10 recovery counts was reported in Table 3 (maximum 10). Supplementary Figure S2 shows the per-template correlation distributions. The _multi regimes used arithmetic-mean aggregation only.

The 1AO7 cohort, which was included in the four-template summary, was also reported separately as the detailed example in Table 4 and Supplementary Tables S19,20. This template contributed 180 variants to the full benchmark (41%), of which n=126 carried at least one CDR mutation. The same Spearman correlation, top- K recovery, and top- K precision calculations were applied under all five structure-input regimes defined in Section 4.3.

## Data Availability

Publicly available datasets were analyzed in this study. This data can be found here: https://atlas.ibbr.umd.edu/web/index.php. The analysis code can be found here: https://github.com/zguo235/tcr_af3.
