# Supplementary material for: Mutational binding assessment for TCR engineering on experimental and AlphaFold 3 scaffolds
Source: Front Bioinform. 2026 Oct 29;6:1879274. doi: 10.3389/fbinf.2026.1879274 (PMC13755275; doi:10.3389/fbinf.2026.1879274)
Supplement: Supplementary file 1 [file DataSheet1.pdf]

Supplementary Material

1 SUPPLEMENTARY TABLES

This document contains inline Supplementary Tables S1–S3 and S21–S22. Supplementary Tables S4–S20 are provided as separate CSV files alongside this PDF.

Table S1. Performance on the SKEMPI v2 dataset for the four state-of-the-art structure-based $\Delta\Delta G$ predictors evaluated in this work, as reported by the original publications (Luo et al., 2023; Wu et al., 2024; Jiao et al., 2025; Xu et al., 2025). All four models use the same evaluation protocol established by Luo et al. (2023): 3-fold cross-validation with the data split by structure, so each fold contains unique protein complexes that do not appear in the other two folds. RMSE and MAE are in kcal/mol. The sequence-only reference predictor PPAC (Zhang et al., 2025) is listed for context but is not protocol-matched: its published figures come from the authors' 5-fold protein-disjoint cross-validation split rather than the 3-fold structure-based split used for the four rows above, and are therefore not directly comparable with those results (†). MAE was not reported.

Method	Year	Pearson	RMSE	MAE
RDE-PPI	2023	0.6447	1.5799	1.1123
Prompt-DDG	2024	0.6772	1.5207	1.0770
BA-DDG	2025	0.7118	1.4516	1.0151
H3-DDG	2025	0.7501	1.3665	0.9612
PPAC (sequence-only)	2025	0.860†	0.732†	—

Table S2. Cα RMSD (Å) between bound and unbound TCR structures for each CDR loop and the framework region, computed after superposition on framework region Cα atoms. Statistics are reported across all 30 matched TCR pairs.

Region	Mean	Std	Min	Max
CDR1α	1.48	0.75	0.32	2.95
CDR2α	1.25	0.57	0.47	2.61
CDR3α	2.22	1.23	0.68	4.93
CDR1β	1.00	0.54	0.28	2.22
CDR2β	1.00	0.65	0.25	3.81
CDR3β	1.69	0.74	0.86	3.88
Framework	0.86	0.24	0.43	1.26
CDR (all)	1.77	0.56	0.84	2.83

Table S3. Per-protein Cα RMSD (Å) of the top-ranked AlphaFold Server prediction compared to the experimental TCR–pMHC structure for each of the 30 TCRFlexDock complexes. CDR and framework values are computed after superposition on TCR framework Cα atoms; the Complex Cα column reports the whole TCR–pMHC complex Cα RMSD after the same superposition. These are the per-protein values that underlie the AlphaFold column block of Table 1 of the article.

PDB	Framework	CDR1α	CDR2α	CDR3α	CDR1β	CDR2β	CDR3β	CDR (all)	Complex Cα
1AO7	0.73	0.37	0.67	0.55	0.47	0.47	1.32	0.84	0.80
1MI5	0.73	0.54	0.41	2.23	0.75	0.27	0.71	1.30	2.36
1MWA	0.70	0.89	0.52	0.74	0.32	0.18	0.76	0.65	1.35
1OGA	0.94	0.69	0.54	0.54	0.33	0.66	0.37	0.52	0.91
2BNR	0.63	0.52	0.38	0.60	0.57	0.49	0.35	0.49	1.17
2CKB	0.70	2.48	0.80	1.42	0.69	0.83	1.38	1.40	4.46
2IAM	0.75	3.32	0.98	1.09	0.34	0.36	0.68	1.38	2.53
2IAN	0.52	2.99	0.91	0.88	0.32	0.36	0.46	1.21	1.31
2NX5	0.88	0.88	0.76	0.93	0.65	0.49	0.47	0.72	1.96
2OI9	0.59	0.48	0.46	0.30	0.38	0.18	0.57	0.42	0.89
2PXY	0.67	0.93	0.83	0.93	0.56	0.31	0.48	0.72	2.54
2PYE	0.76	0.15	0.60	0.33	0.21	0.43	0.23	0.35	0.73
3DXA	1.04	1.72	0.83	1.38	0.84	0.63	1.62	1.34	1.40
3H9S	0.64	0.51	0.57	0.37	0.30	0.67	0.86	0.62	1.12
3KPR	0.63	0.70	0.58	4.62	0.71	0.33	0.69	2.55	1.55
3KPS	0.51	0.66	0.56	2.06	0.75	0.34	0.94	1.27	1.47
3PWP	0.51	0.60	0.61	0.36	0.32	0.77	0.65	0.58	1.17
3QDG	0.77	0.71	0.35	0.93	0.38	0.42	0.57	0.64	0.93
3QDJ	0.77	0.44	0.39	0.55	0.22	0.24	0.53	0.45	0.94
3SJV	1.32	1.07	1.34	1.04	0.70	0.99	0.69	0.97	1.41
3UTT	0.86	0.57	0.78	0.67	0.94	0.53	0.82	0.74	1.77
3VXS	0.61	0.50	0.83	0.44	0.61	0.84	0.42	0.60	2.98
3W0W	1.72	2.68	1.34	1.27	1.27	1.23	1.04	1.51	1.60
4JFD	1.00	0.28	0.46	0.52	0.49	0.27	0.37	0.41	0.87
5NMF	0.65	0.64	0.46	0.42	0.51	0.58	0.43	0.50	0.94
5NMG	0.81	0.57	0.54	0.77	0.61	0.53	0.64	0.64	1.22
6AVF	0.99	0.79	0.93	1.09	0.31	0.56	0.52	0.79	3.50
6CQL	0.58	0.61	0.78	0.52	0.58	0.50	0.63	0.61	1.78
6CQQ	0.71	0.41	0.51	0.36	0.33	0.58	0.34	0.42	1.84
6CQR	0.63	1.01	0.67	0.50	0.47	0.78	2.05	1.23	1.49

2 EXTERNAL SUPPLEMENTARY TABLES S4–S20

The following additional CSV files accompany this supplementary PDF and contain data that is too large for inline presentation:

- **S4.csv** — Per-complex all-heavy-atom and side-chain RMSD comparisons for 30 experimental bound–unbound TCR pairs and 30 comparisons between top-ranked AF3 predictions and experimental bound structures (60 rows). Structures were aligned on framework Cα atoms; metrics include framework, pooled CDR, individual CDR-loop, and experimental-reference 5-Å interface regions, atom and residue counts, and residue-mismatch exclusions.
- **S5.csv** — Residue-wise all-heavy-atom and side-chain RMSD values for the same 60 comparisons, indexed by TCR chain, IMGT position, region, residue identity, and experimental-reference interface membership (13,168 rows).
- **S6.csv** — The 438-mutant ATLAS evaluation cohort with the full ATLAS metadata schema, after the cleaning pipeline described in §4.3 of the article.
- **S7–S9.csv** — Per-template Pearson r (S7), RMSE (S8), and MAE (S9) for the four structure-based $\Delta\Delta G$ models across the five structure-input regimes of Table 2 of the article. One row per template (37 rows $\times$ 22 columns).
- **S10.csv** — Prediction-accuracy estimates and 95% confidence intervals for Pearson r, RMSE, and MAE for the 36 structure-based predictor–input combinations in Table 2 and the sequence-only PPAC baseline (37 rows; 10,000 bootstrap resamples of the $n = 438$ variants).
- **S11.csv** — Single-versus-arithmetic-mean-ensemble Pearson comparisons for the four models on AF3-WT and AF3-mutant scaffolds (eight comparisons): individual correlations and 95% confidence intervals, Δr and its 10,000-replicate paired-bootstrap interval, Williams-test statistic, degrees of freedom, and two-sided p-value.
- **S12.csv** — Per-template Pearson r and 10,000-replicate within-template bootstrap 95% confidence intervals for all 37 templates, four structure-based predictors, and five structural-input regimes (740 rows). Intervals are reported for templates with at least four mutations; cells with $n < 4$ are marked `not_estimable_n_lt_4`.
- **S13.csv** — Mutant-AF3 ensemble-size saturation results for a randomly selected cohort of 100 mutations: Pearson r, RMSE, and MAE for mean, median, and AF3-ranking-score-weighted aggregation at $N = 1, 10, 20, 50, 100$, together with 10,000-replicate paired-bootstrap confidence intervals for each metric and for its difference from $N = 100$ (180 rows).
- **S14.csv** — The randomly selected 100-mutation ATLAS cohort used for the saturation analysis, including template, mutation, experimental $\Delta\Delta G$, and selection order (100 rows).
- **S15.csv** — One row for each of the 37 AF3-WT templates, containing sequence-alignment coverage; 4-, 5-, and 6-Å TCR–pMHC contact agreement; framework-superposed CDR3 and whole-complex RMSD; pTM, ipTM, ranking score, interface PAE/contact probability, regional pLDDT, and other AF3 confidence metrics; per-model PDB and AF3-WT mean absolute errors; and mean absolute-error changes.
- **S16.csv** — One row for each of the 438 mutations, containing mutation region and normalized peptide position; mutation-local 4-, 5-, and 6-Å contact agreement; mutation-site pLDDT and partner PAE; experimental and predicted $\Delta\Delta G$; PDB and AF3-WT absolute errors; per-model absolute-error

changes; and pairwise differences in absolute-error change between BA-DDG/Prompt-DDG and H3-DDG/Prompt-DDG.

- **S17.csv** — Long-form results for the AF3-WT structural error-feature analysis: prespecified and exploratory template-level associations, equal-template within-template mutation associations, model contrasts, contact-cutoff and minimum-template-size sensitivity analyses, categorical means, 95% template-block bootstrap intervals, permutation p-values, and Benjamini–Hochberg-adjusted q-values (355 rows).
- **S18.csv** — Per-template candidate-prioritization metrics for four ATLAS templates, each with at least 20 measured variants carrying at least one CDR mutation (1AO7, 1MI5, 2BNR, and 3QDJ; combined $n = 191$): per (template, model, regime) Spearman ρ, 10,000-replicate within-template bootstrap 95% confidence intervals, and top-K precision ($K = 3, 5, 10$). One row per (template, model, regime) combination (80 rows). Provides the per-template data underlying Table 3 of the article and Supplementary Figure S2.
- **S19.csv** — Candidate-prioritization metrics for the $n = 126$ 1AO7 variants carrying at least one CDR mutation, analyzed in §2.5 of the article: per model–regime combination Spearman ρ and top-K precision ($K = 3, 5, 10$).
- **S20.csv** — Per-mutation predicted ranks (1–126) of the 10 most-stabilizing 1AO7 CDR mutants under each of the 4 models $\times$ 5 regimes.

2.1 Confidence-Interval Results

Complete confidence intervals for prediction accuracy, single-versus-ensemble comparisons, candidate prioritization, AF3-WT error-feature associations, ensemble-size saturation, and per-template accuracy are provided in Supplementary Tables S10–S13 and S17–S18. The sequence-only PPAC reference had Pearson $r = 0.504$ (95% CI, 0.432–0.572) on the 438-variant cohort (Supplementary Table S10). Per-complex values are provided in Supplementary Table S4.

3 ROBUSTNESS TO PREDICTED SCAFFOLDS BY SKEMPI TEMPLATE OVERLAP

3.1 Methods

We assessed whether sensitivity to replacing experimental structures with predicted scaffolds differed according to template overlap between the predictor training set SKEMPI 2.0 and test set ATLAS. Overlapped templates were defined by matching four-character template PDB IDs. Of the 37 unique ATLAS template IDs, 15 also occurred among the 345 unique SKEMPI template IDs. The shared-PDB-ID group contained 356 ATLAS records across 15 templates, and the no-shared-PDB-ID group contained 82 records across 22 templates.

RDE-PPI and Prompt-DDG were compared using experimental WT structures and the top-ranked AF3 prediction of each WT complex (AF3-WT single). Pearson r, root-mean-square error (RMSE), and mean absolute error (MAE) were calculated. Scaffold sensitivity was quantified as AF3-WT single minus experimental WT performance on the same records: negative Δr and positive ΔRMSE or ΔMAE indicate deterioration. Error metrics and their changes are reported in kcal/mol. Percentile 95% confidence intervals were obtained from 10,000 bootstrap resamples. Between-group contrasts were defined as the change in the no-shared-ID group minus that in the shared-ID group.

3.2 Results

Both predictors had lower point estimates of Pearson r in the no-shared-PDB-ID group under both structural inputs (Table S21A). On experimental WT structures, Pearson r was 0.854 for RDE-PPI and 0.842 for Prompt-DDG in the shared-ID group, compared with 0.744 and 0.686, respectively, in the no-shared-ID group. On AF3-WT single structures, Pearson r was 0.795 for RDE-PPI and 0.821 for Prompt-DDG in the shared-ID group, compared with 0.717 and 0.617, respectively, in the no-shared-ID group. Prompt-DDG shows a smaller correlation loss in the shared-ID group; RDE-PPI shows a smaller loss in the no-shared-ID group. The same ordering holds for the RMSE and MAE changes (Table S21B).

The between-group differences in correlation change were 0.032 (95% CI, $[-0.041,\ 0.126]$) for RDE-PPI and -0.048 ($[-0.281,\ 0.047]$) for Prompt-DDG; both intervals included zero (Table S22). RDE-PPI's MAE increase was 0.221 kcal/mol in the shared-ID group and 0.029 kcal/mol in the no-shared-ID group, giving a between-group difference of -0.193 kcal/mol ($[-0.291,\ -0.036]$). For Prompt-DDG, the between-group difference in MAE change was -0.060 kcal/mol ($[-0.127,\ 0.044]$).

Fifteen of the 37 ATLAS template PDB IDs were present in SKEMPI 2.0. On AF3-WT inputs, Prompt-DDG had higher Pearson r and lower RMSE and MAE point estimates than RDE-PPI in the shared-ID group, whereas this ordering was reversed in the no-shared-ID group (Table S21A). Shared-template status did not consistently correspond to smaller performance losses on AF3-WT: RDE-PPI's MAE increase was larger in the shared-ID group, while the between-group differences in correlation change for both predictors and MAE change for Prompt-DDG remained uncertain (Table S22). Training overlap may contribute to the differences in accuracy, but this comparison cannot distinguish its effects on the robustness to predicted scaffolds.

Table S21. Prediction accuracy and changes following replacement of experimental WT structures with AF3-WT single structures, stratified by template PDB-ID overlap with SKEMPI 2.0. The shared-ID group contains 356 ATLAS records on 15 templates; the no-shared-ID group contains 82 records on 22 templates. Panel A reports pooled Pearson r, RMSE, and MAE. Panel B reports AF3-WT single minus experimental WT performance on the same records: negative Δr and positive error changes indicate deterioration. Error metrics and their changes are in kcal/mol. Brackets contain percentile 95% confidence intervals from 10,000 bootstrap resamples.

A. Prediction accuracy

Predictor	Structural input	Pearson r	RMSE	MAE
**Shared PDB ID: 356 records, 15 templates**				
RDE-PPI	Experimental WT	0.854 [0.655, 0.920]	1.232 [0.745, 2.124]	0.765 [0.449, 1.459]
RDE-PPI	AF3-WT single	0.795 [0.598, 0.908]	1.462 [0.961, 2.236]	0.986 [0.604, 1.619]
Prompt-DDG	Experimental WT	0.842 [0.646, 0.937]	1.295 [0.682, 2.426]	0.739 [0.448, 1.639]
Prompt-DDG	AF3-WT single	0.821 [0.633, 0.917]	1.403 [0.803, 2.500]	0.839 [0.517, 1.725]
**No shared PDB ID: 82 records, 22 templates**				
RDE-PPI	Experimental WT	0.744 [0.124, 0.879]	1.331 [0.983, 1.719]	0.993 [0.711, 1.368]
RDE-PPI	AF3-WT single	0.717 [0.111, 0.859]	1.385 [0.964, 1.830]	1.022 [0.700, 1.473]
Prompt-DDG	Experimental WT	0.686 [−0.001, 0.877]	1.540 [0.906, 2.145]	1.047 [0.629, 1.632]
Prompt-DDG	AF3-WT single	0.617 [−0.143, 0.867]	1.627 [0.901, 2.304]	1.087 [0.647, 1.713]

B. Changes from experimental WT to AF3-WT single

Predictor	Δr	ΔRMSE	ΔMAE
**Shared PDB ID: 356 records, 15 templates**			
RDE-PPI	−0.059 [−0.136, −0.004]	0.230 [0.013, 0.359]	0.221 [0.119, 0.265]
Prompt-DDG	−0.021 [−0.053, −0.001]	0.108 [−0.004, 0.186]	0.100 [0.026, 0.130]
**No shared PDB ID: 82 records, 22 templates**			
RDE-PPI	−0.027 [−0.071, 0.032]	0.054 [−0.075, 0.180]	0.029 [−0.059, 0.139]
Prompt-DDG	−0.069 [−0.299, 0.020]	0.087 [−0.015, 0.220]	0.040 [−0.018, 0.117]

Table S22. Between-group differences in the changes from experimental WT to AF3-WT single performance. Each contrast is the change in the no-shared-PDB-ID group minus the change in the shared-PDB-ID group. Negative correlation-change contrasts and positive error-change contrasts indicate greater deterioration in the no-shared-ID group. Error-change contrasts are in kcal/mol. Brackets contain percentile 95% confidence intervals from 10,000 bootstrap resamples drawn independently within the two groups.

Predictor	Difference in Δr	Difference in ΔRMSE	Difference in ΔMAE
RDE-PPI	0.032 [−0.041, 0.126]	−0.176 [−0.364, 0.078]	−0.193 [−0.291, −0.036]
Prompt-DDG	−0.048 [−0.281, 0.047]	−0.021 [−0.157, 0.147]	−0.060 [−0.127, 0.044]

4 SUPPLEMENTARY FIGURES

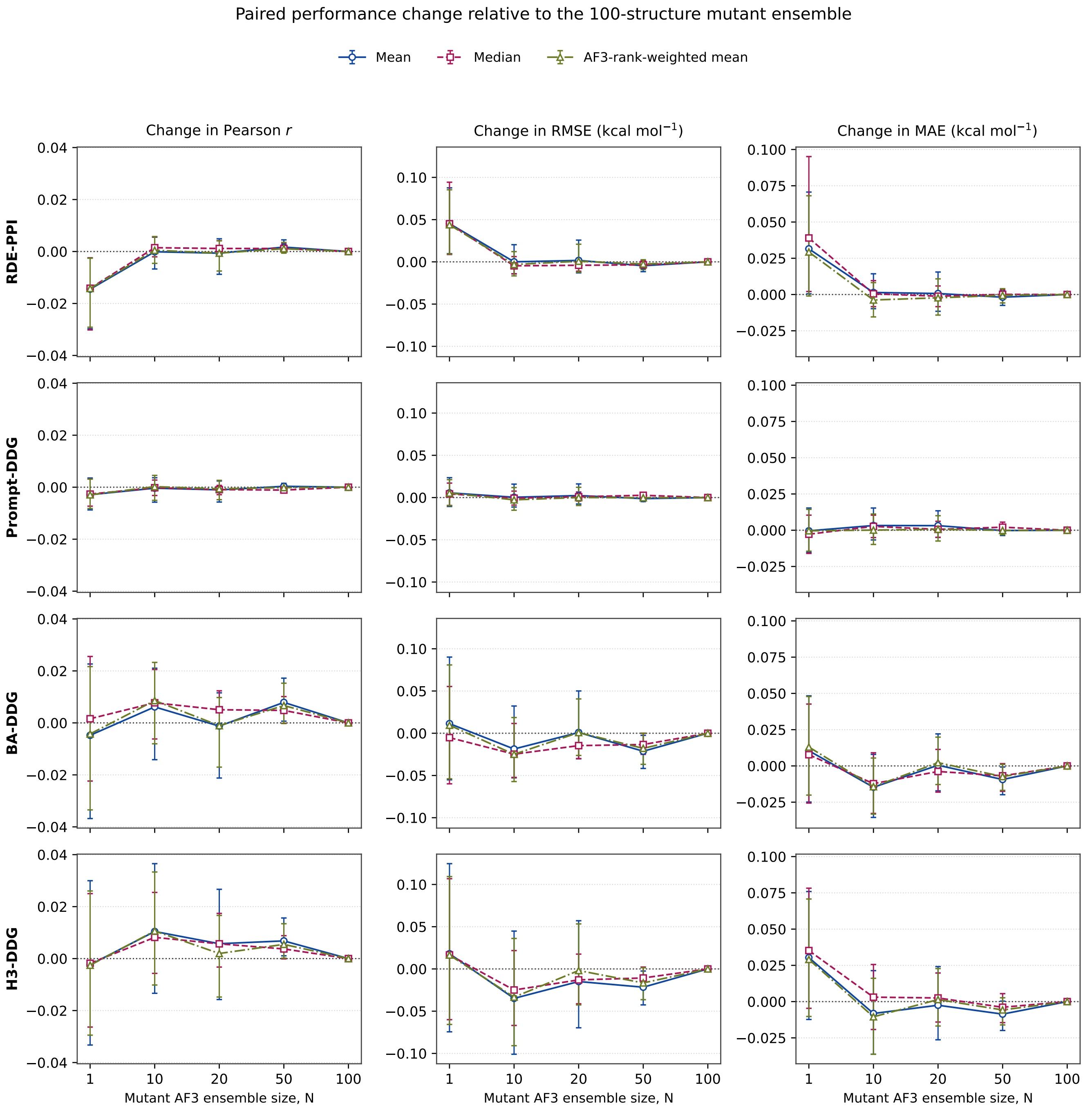

Figure S1. Saturation of mutant-specific AF3 ensemble aggregation for a randomly selected cohort of 100 ATLAS mutants. Each panel shows the paired change in downstream performance at ensemble size N relative to the full $N = 100$ ensemble for one predictor, aggregation rule, and metric; points are observed differences and bars are 95% paired-bootstrap confidence intervals (10,000 resamples). The horizontal reference line marks no change from $N = 100$. All 36 intervals at $N = 20$ include zero; the largest absolute $N = 20$ difference is 0.006 for Pearson r, 0.015 kcal/mol for RMSE, and 0.004 kcal/mol for MAE. These results show no detectable downstream-performance improvement beyond $N = 20$ within this cohort.

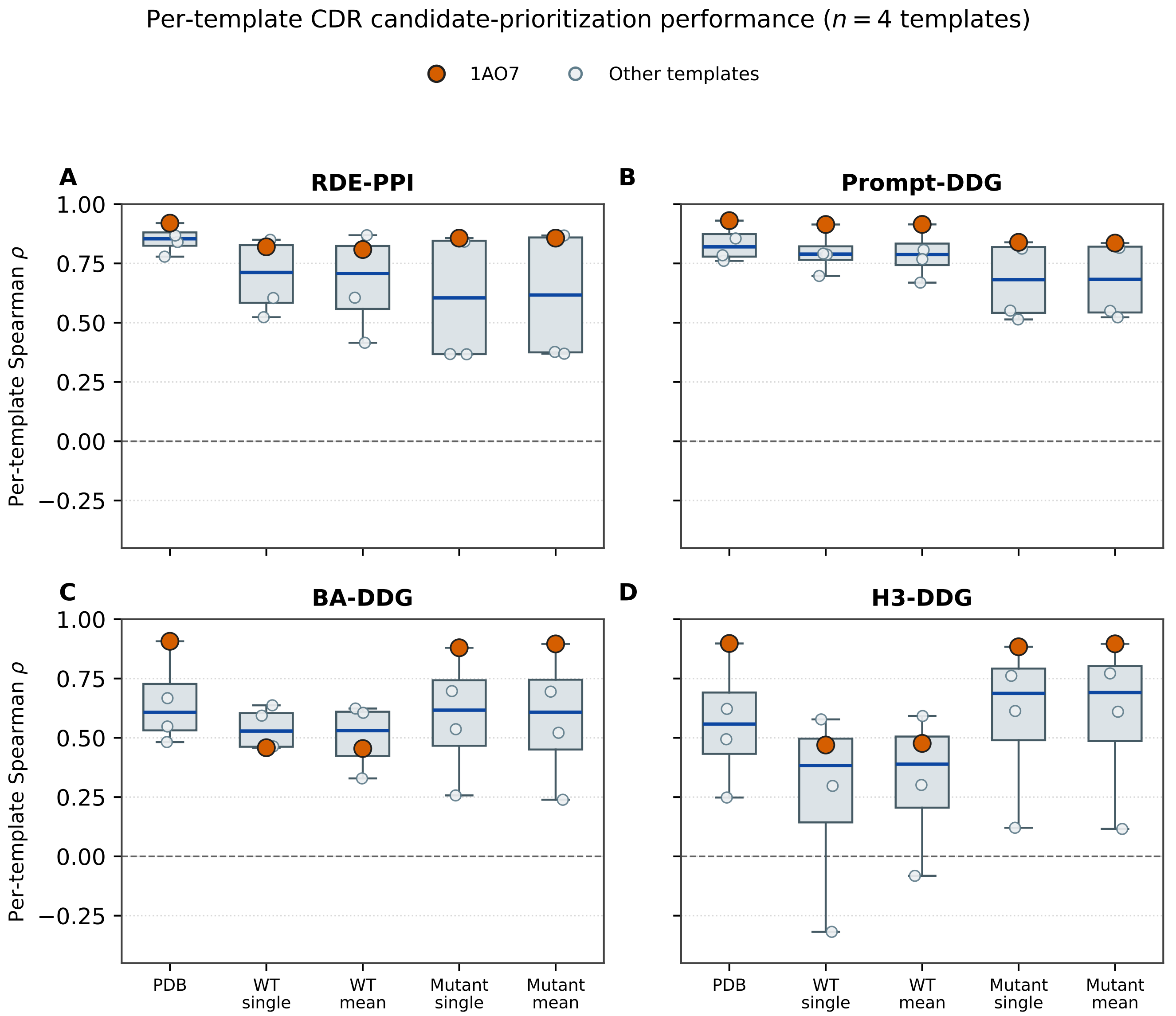

Figure S2. Per-template Spearman ρ distributions for candidate prioritization across four ATLAS templates, each with at least 20 measured variants carrying at least one CDR mutation (1AO7, 1MI5, 2BNR, and 3QDJ; combined $n = 191$). Each panel corresponds to one $\Delta\Delta G$ predictor, and the five x-axis positions are the structure-input settings reported in Table 3 of the article. Boxes show the median and IQR across the four templates, with whiskers extending to the data range. Per-template values are overlaid as open gray circles for the three other templates and a larger filled orange circle with a dark outline for 1AO7. The dashed horizontal line marks $\rho = 0$. The `_multi` settings use arithmetic-mean aggregation across AF3 samples; per-template values and 95% CIs are provided in Supplementary Table S18.

REFERENCES

Jiao, X., Mao, W., Jin, W., Yang, P., Chen, H., and Shen, C. (2025). Boltzmann-aligned inverse folding model as a predictor of mutational effects on protein-protein interactions. In *The Thirteenth International Conference on Learning Representations*

Luo, S., Su, Y., Wu, Z., Su, C., Peng, J., and Ma, J. (2023). Rotamer density estimator is an unsupervised learner of the effect of mutations on protein-protein interaction. In *The Eleventh International Conference on Learning Representations*

Wu, L., Tian, Y., Lin, H., Huang, Y., Li, S., Chawla, N. V., et al. (2024). Learning to predict mutational effects of protein-protein interactions by microenvironment-aware hierarchical prompt learning. In *Proceedings of the 41st International Conference on Machine Learning* (PMLR), 53847–53859

Xu, D., Rao, J., Zhu, M., Zhang, J., Lu, W., Zheng, S., et al. (2025). Accurately predicting protein mutational effects via a hierarchical many-body attention network. In *The Thirty-ninth Annual Conference on Neural Information Processing Systems*

Zhang, L., Zhou, X., Liang, L., and Lin, J. (2025). PPAC: Predicting protein–protein affinity changes induced by amino acid mutations using protein large language models. *ACS Omega* 10, 54895–54902. doi:10.1021/acsomega.5c08777
